# The G protein‐coupled receptor GPR89A is a novel potential therapeutic target to overcome cisplatin resistance in NSCLC Calu1 cells

**DOI:** 10.1111/febs.70099

**Published:** 2025-04-17

**Authors:** Hale Guler Kara, Eda Dogan, Vildan Bozok, Cagdas Aktan, Ece Cakiroglu, Zuhal Eroglu, Duygu Aygunes Jafari, Kemal Sami Korkmaz, Serif Senturk, Buket Kosova

**Affiliations:** ^1^ Department of Medical Biology Ege University School of Medicine Izmir Turkey; ^2^ Department of Medical Biology Harran University School of Medicine Sanliurfa Turkey; ^3^ Department of Medical Biology Bandirma Onyedi Eylul University School of Medicine Balikesir Turkey; ^4^ Izmir Biomedicine and Genome Center Turkey; ^5^ Izmir International Biomedicine and Genome Institute Dokuz Eylul University Izmir Turkey; ^6^ Department of Bioengineering Ege University School of Engineering Izmir Turkey

**Keywords:** cisplatin resistance, CRISPR‐Cas9, genetic screen, *GPR89A*, NSCLC

## Abstract

Lung cancer is the most frequently diagnosed cancer type worldwide and is characterised by its high metastatic potential. Standard therapy for nonsmall cell lung cancer (NSCLC) cases includes chemotherapy with the platinum‐based chemotherapeutic agent cisplatin. Although lung cancer cases respond well to cisplatin at the beginning of treatment, ~ 60% develop chemotherapy resistance during this process. In this study, a genome‐wide CRISPR‐Cas9‐based genetic screening approach was employed to identify genes that cisplatin‐resistant NSCLC Calu1 cells are more addicted to than sensitive cells. Cisplatin‐resistant Calu1 cells were generated by the dose escalation method, and genome‐wide CRISPR‐Cas9‐based genetic screening was performed with the Brunello CRISPR knockout library. Bioinformatics analyses of the obtained next‐generation sequencing data revealed 63 potential candidate genes responsible for cisplatin resistance, including *G protein‐coupled receptor 89A* (*GPR89A*), *Poly(U) binding splicing factor 60* (*PUF60*), *NBAS subunit of NRZ tethering complex* (*NBAS*) and *GrpE like 1*, *mitochondrial* (*GRPEL1*). The GPR89A protein is located in the Golgi cisterna and Golgi‐associated vesicle membrane, enables voltage‐gated anion channel activity, and is involved in intracellular pH reduction. Functional studies carried out with *GPR89A*‐knockout cisplatin‐resistant Calu1 cells resulted in cell cycle arrest in the G2/M phase and increased polyploidy, and also prevented colony formation and cell migration. Cisplatin treatment, on the other hand, resulted in increased cell death by apoptosis upon cell cycle arrest in the S phase. In conclusion, this is the first study that identified *GPR89A* as a potential therapeutic target to overcome cisplatin resistance in NSCLC Calu1 cells.

## Graphical abstract

A genome‐wide CRISPR‐Cas9‐based genetic screen in cisplatin‐resistant NSCLC cells revealed 63 potential candidate genes for overcoming cisplatin resistance, including *GPR89A*. *GPR89A*, localised in the Golgi, modulates voltage‐gated anion channel activity and is involved in intracellular pH reduction. Here, we identified *GRPR89A* as a previously undescribed therapeutic target in NSCLC cells and found that cisplatin treatment in *GPR89A* knockout cells increased cell death following cell cycle arrest in the S phase.

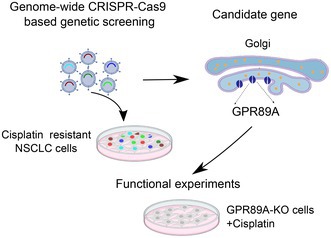

**Abbreviations**
ARIH1: ariadne RBR E3 ubiquitin protein ligase 1
AURKA: aurora kinase A
BCL2L1: BCL2‐like 1
Cas9: CRISPR associated protein 9
CRISPR: clustered regularly interspaced short palindromic repeats
DGKD: diacylglycerol kinase delta
EGFR: epidermal growth factor receptor
FBS: fetal bovine serum
GPR89A: G protein‐coupled receptor 89A
GRPEL1: GrpE like 1, mitochondrial
MAP3K12: mitogen‐activated protein kinase kinase kinase 12
MOI: multiplicity of virus infection
MPP3: MAGUK p55 scaffold protein 3
NBAS: NBAS subunit of NRZ tethering complex
NF2: moesin‐ezrin‐radixin‐like (MERLIN) tumour suppressor
NGS: next‐generation sequencing
NSCLC: nonsmall cell lung cancer
POTEC: POTE ankyrin domain family member C
PUF60: poly(U) binding splicing factor 60
SCLC: small cell lung cancer
SEMA4G: semaphorin 4G
SLC35A2: solute carrier family 35 member A2
SPEG: striated muscle enriched protein kinase
SULF1: sulfatase 1
TADA1: transcriptional adaptor 1
USP17L20: ubiquitin‐specific peptidase 17 like family member 20
WEE1: WEE1 G2 checkpoint kinase
ZNF587B: zinc finger protein 587B
ZNRF3: zinc and ring finger 3

## Introduction

Lung cancer is the leading cause of cancer‐related deaths due to its rapid progression and high metastatic potential, accounting for 11.7% of all newly diagnosed malignancies and 18% of all deaths [1, 2]. The two main types of lung cancer are small cell lung cancer (SCLC) and nonsmall cell lung cancer (NSCLC), with NSCLC diagnosed in ~ 85% of all cases [3]. Numerous environmental and genetic factors are involved in its development and progression, unveiling malignant hallmarks such as uncontrolled cell proliferation, invasion, metastasis, and drug resistance [4, 5]. Cancer cells are more addicted to certain genetic factors than normal cells, because of their high mutational burden; wherein the strength of chemotherapy comes into play to repress the effectiveness of such factors. Cisplatin is a platinum‐based chemotherapeutic agent widely used in the treatment of various solid tumours and in particularly NSCLC [5]. Cisplatin exerts its anticancer activity by causing the formation of intra‐ and inter‐chain cross‐links through its interaction with purine bases in the DNA. This cisplatin‐DNA adducts further prevent replication and transcription by bending the DNA duplex and induce apoptotic cell death by causing cell cycle arrest [6, 7, 8]. Although many cancer cells are initially sensitive to cisplatin treatment, changes in drug uptake and detoxification, increased DNA damage repair or inhibition of apoptotic cell death can eventually lead to chemotherapy resistance [9]. Overcoming cisplatin resistance is an important goal in NSCLC treatment, yet many genes and biological processes involved in its development are still unknown. Therefore, new strategies have to be exploited to fulfil this criterion.

CRISPR‐Cas9‐based genetic screenings were initially developed as a gene‐editing tool derived from the prokaryotes native immune defence system [10, 11]. Later, they were also adapted to many other fields such as gene deletion/insertion, base editing, expressional/epigenetic regulation, imaging, diagnostic testing and functional genetic screening [12]. So far, CRISPR‐Cas9‐based genetic screenings have been widely employed for the determination of disease‐causing genetic factors and factors involved in disease progression [13, 14]. Nonetheless, similar screenings can be also applied for the discovery of genes and biological processes involved in chemotherapy resistance [15, 16].

The aim of this study, therefore, was to determine candidate genes that cisplatin‐resistant NSCLC Calu1 cells are more addicted to than sensitive cells by a genome‐wide CRISPR‐Cas9‐based genetic screening approach and to evaluate them as therapeutic targets to resensitise cells.

## Results

### Characterisation of CR‐Calu1 cells

**Fig. 1:**
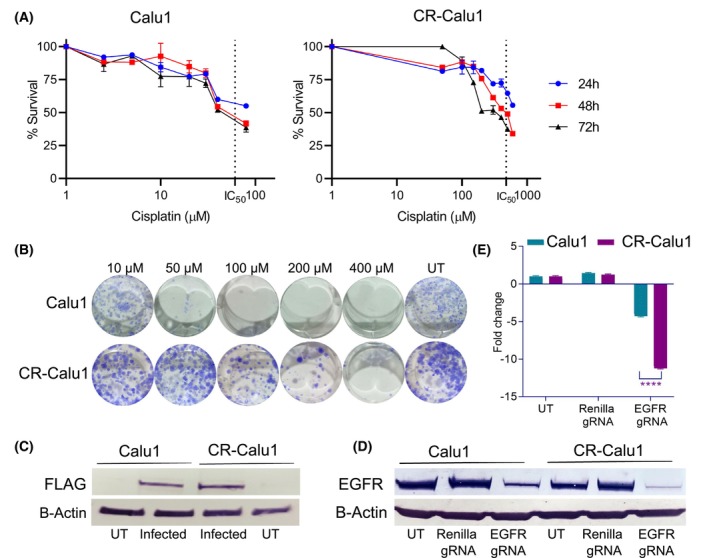
Characterisation of cisplatin resistance and functional Cas9 expression. (A) Cell viability graph of Calu1 and cisplatin‐resistant Calu1 cells (CR‐Calu1) at 24, 48 and 72 h. Calu1 cells were treated with 2.5–5–10–20–30–40–80 μm cisplatin; and, CR‐Calu1 cells with 50–100–150–200–300–400–500–600 μm cisplatin, in triplicates. The IC_50_ doses for cisplatin were determined from the dose–response curves of nonlinear regression analysis using graphpad prism 8.0.2 software. The 24‐, 48‐ and 72‐h IC_50_ values of parental and resistant cells were 83.49, 69.66 and 54.35 μm for Calu1; 821.4, 478.8 and 347.1 μm for CR‐Calu1 cells, respectively; IC_50_ values for 48 h are shown in the plots. (B) Colony formation of Calu1 and CR‐Calu1 cells. Cells were untreated (UT) or treated with the indicated cisplatin concentrations for 48 h; experiments were performed in triplicates; (C) FLAG‐tagged Cas9 expression. Cells were transduced (infected) with the LentiCas9‐Blast vector, selected for 9 days with blasticidin and grown for another 14 days with normal medium. Protein lysates were obtained, and western blot analyses performed with the anti‐FLAG antibody; triplicates. (D) EGFR protein expression. Cas9 expressing cells were untreated (UT) or transduced with either the *Renilla reniformis* luciferase (*RLuc*) gRNA or epidermal growth factor receptor (EGFR) gRNA, before western blot analyses were performed with the anti‐EGFR antibody; experiments were performed in triplicates. (E) EGFR protein expression fold change normalised to β‐actin. EGFR protein expression levels decreased 4.27‐ and 11.22‐fold in EGFR gRNA transduced Calu1 and CR‐Calu1 cells, respectively, when compared to *RLuc* gRNA transduced cells; experiments were performed in triplicates. The statistical analysis was conducted using Student's *t*‐test (*P* < 0.0001). Protein expression levels were determined using the imagej software (error bars indicate standard deviation).

Cisplatin‐resistant Calu1 (CR‐Calu1) cells were generated by the dose escalation method, that is, exposing cisplatin‐sensitive Calu1 cells to increasing concentrations of cisplatin. IC_50_ values obtained for Calu1 cells treated with cisplatin concentrations ranging from 2.5 to 80 μm were 83.49 μm at 24 h, 69.66 μm at 48 h, and 54.35 μm at 72 h. However, IC_50_ values obtained for CR‐Calu1 cells treated with cisplatin concentrations ranging from 50 to 600 μm were 821.4 μm at 24 h, 478.8 μm at 48 h and 347.1 μm at 72 h (Fig. 1A). According to this, CR‐Calu1 cells were 9.8‐fold more resistant to cisplatin at 24 h, 6.9‐fold at 48 h and 6.3‐fold at 72 h. In fact, CR‐Calu1 cells were resistant up to 400 μm of cisplatin and colony formation continued to a limited extent. In contrast, colony formation of Calu1 cells decreased significantly at 50 μm of cisplatin and colonies did not form at subsequent increasing doses (Fig. 1B). Stable expression of FLAG‐tagged Cas9 protein in LentiCas9‐Blast transduced Calu1 and CR‐Calu1 cells was demonstrated by western blot analyses (Fig. 1C). Functional Cas9 protein expression was also demonstrated by western blot analyses comparing EGFR expression levels after transducing Calu1 and CR‐Calu1 cells either with *RLuc* or *EGFR* gRNA‐expressing lentiviruses. EGFR protein expression levels decreased 4.27‐ and 11.22‐fold in *EGFR* gRNA transduced Calu1 and CR‐Calu1 cells, respectively, when compared to *RLuc* gRNA transduced cells (Fig. 1D,E). Lentivirus transduction alone had no cytotoxic effect on cells.

### Genome‐wide CRISPR‐Cas9‐based genetic screening identified novel candidate genes involved in cisplatin resistance

**Fig. 2:**
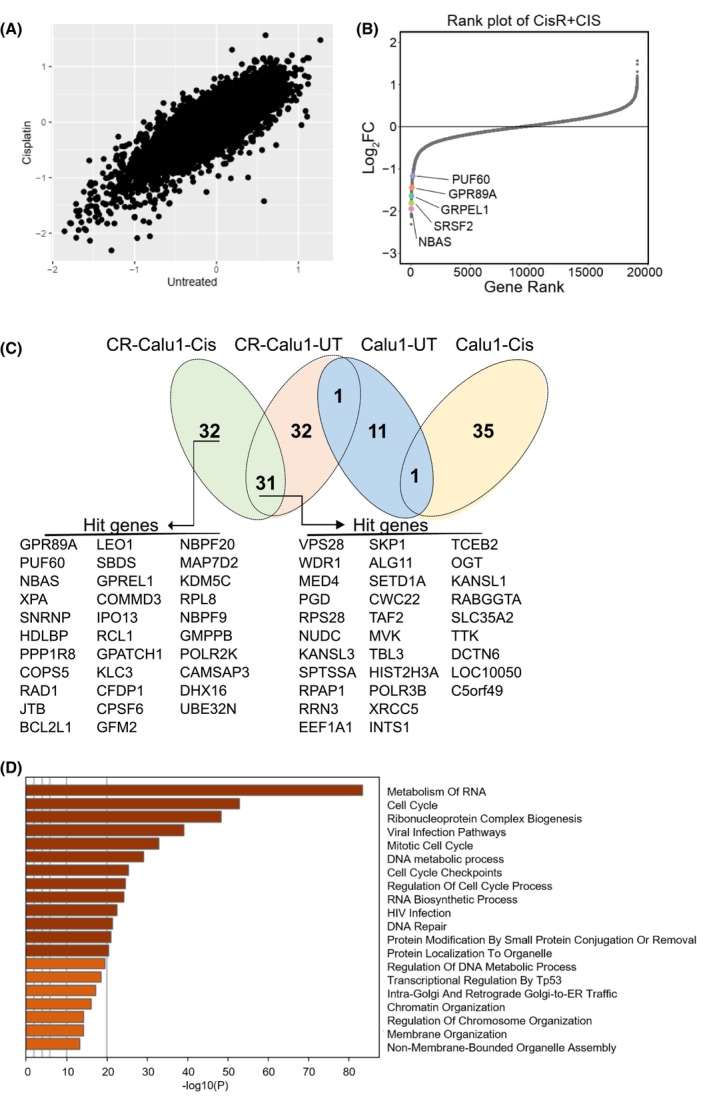
Genome‐wide CRISPR‐Cas9‐based genetic screening identified novel candidate genes involved in cisplatin resistance in NSCLC cells. (A) Log‐fold changes of genes after CRISPR‐Cas9‐based genetic screening in cisplatin‐resistant Calu1 cells (CR‐Calu1); experiments were performed in triplicates. (B) LogFC distribution of hit genes identified in cisplatin‐treated and cisplatin‐untreated CR‐Calu1 cells; analyses were performed in triplicates. (C) Hit gene list created from genes that were depleted through negative selection in each cell group (Cis, cisplatin‐treated cell group; UT, cisplatin‐untreated cell group). (D) Gene Ontology (GO) analysis of selected hit genes in the CRISPR‐Cas9 screen. Top 20 Kyoto Encyclopedia of Genes and Genomes (KEGG) and GO enriched terms of overlapping genes are represented by different colours, that is the darker the colour, the higher the enrichment. Horizontal axis represents the −log_10_ *P*‐value and vertical axis represents the enriched terms (NSCLC, nonsmall cell lung cancer).

**Fig. 3:**
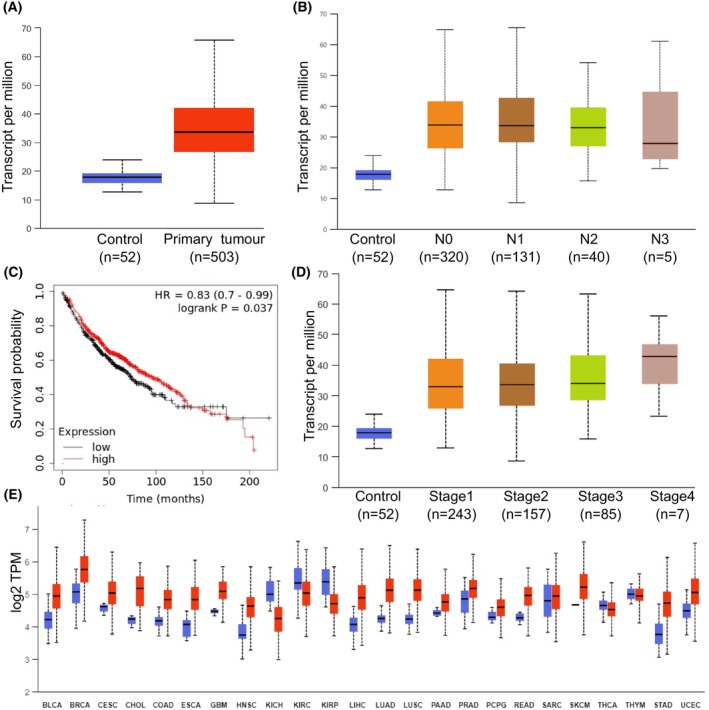
*GPR89A* expression profile in the TCGA database. (A) GPR89A expression profile in normal lung tissue (blue) and primary lung tumour tissue (red); statistical significance was calculated using an independent two‐sample *t*‐test (*P* < 0.05); (B) GPR89A expression profile in lung cancer tissues based on the nodal (N) metastatic status; statistical significance was calculated using an independent two‐sample *t*‐test (*P* < 0.05) (boxplots showing gene expression levels between groups); (C) Effect of GPR89A expression levels on disease‐free survival of 1876 lung cancer cases (*P* < 0.037); (D) GPR89A expression profile in lung cancer tissues based on cancer stages; (E) GPR89A expression profile across different cancer types in The Cancer Genome Atlas Program (TCGA) database [in normal (blue) and cancer (red) tissues; error bars indicate standard deviation]. BLCA, bladder urothelial carcinoma; BRCA, breast invasive carcinoma; CESC, cervical squamous cell carcinoma and endocervical adenocarcinoma; CHOL, cholangiocarcinoma; COAD, colon adenocarcinoma; ESCA, oesophageal carcinoma; GBM, glioblastoma; HNSC, head–neck squamous cell carcinoma; KICH, kidney chromophobe; KIRC, kidney renal clear cell carcinoma; KIRP, kidney renal papillary cell carcinoma; LIHC, liver hepatocellular carcinoma; LUAD, lung adenocarcinoma; LUSC, lung squamous cell carcinoma; PAAD, pancreatic adenocarcinoma; PCPG, pheochromocytoma and paraganglioma; PRAD, prostate adenocarcinoma; READ, rectum adenocarcinoma; SARC, sarcoma; SKCM, skin cutaneous melanoma; STAD, stomach adenocarcinoma; THCA, thyroid cancer; THYM, thymoma; UCEC, uterine corpus endometrial carcinoma.

Genome‐wide CRISPR‐Cas9‐based genetic screening was performed with the lentiviral Brunello CRISPR knockout library consisting of 76.441 gRNAs targeting 19.114 human genes. After transduction, Calu1 and CR‐Calu1 cells were each divided into cisplatin‐treated and cisplatin‐untreated groups. Amplicon sequencing of isolated genomic DNAs was performed by NGS to determine the gRNA distribution and subsequent bioinformatics analyses of the resulting NGS data revealed gRNA abundance for each cell group (Fig. 2A,B). Genes targeted by the relevant gRNAs were listed, and a hit gene list was created from genes that were depleted through negative selection. As a result, 63 candidate genes were identified in cisplatin‐treated CR‐Calu1 cells; 64 candidate genes in untreated CR‐Calu1 cells; 36 candidate genes in cisplatin‐treated Calu1 cells; and 13 candidate genes in untreated Calu1 cells (Fig. 2C). KEGG and GO analyses of the candidate genes revealed the biological pathways they are involved in for developing cisplatin resistance, including DNA repair, cell cycle, chromosome and membrane organisation, ER‐Golgi transport, RNA biosynthetic process, protein transport, ribonucleoprotein complex biogenesis, transcriptional regulation and metabolic processes (Fig. 2D). *GPR89A* was found as one of the candidate genes depleted in CR‐Calu1 cells and encodes for the G protein‐coupled receptor 89A protein located in the Golgi cisterna and Golgi‐associated vesicle membrane. Although its involvement in voltage‐gated anion channel activity and intracellular pH reduction was known, its role in cisplatin resistance in NSCLC cells was new. Analysis of normal lung and primary lung tumour tissues from the TCGA database revealed an increase of *GPR89A* expression in cancerous tissues (Fig. 3A, *P* < 0.05). When nodal metastatic states (N0, N1, N2, N3) and individual cancer stages were compared, again an increase of *GPR89A* expression in all lung cancer tissues could be observed (Fig. 3B,D, *P* < 0.05). Kaplan–Meier survival analyses also revealed that the disease‐free survival rates decreased in 1876 lung cancer cases with increased *GPR89A* expression (Fig. 3C, *P* = 0.037). Increased *GPR89A* expression levels were also found in many other cancer types, for example, breast invasive carcinoma (BRCA), cervical squamous cell carcinoma and endocervical adenocarcinoma (CESC), and cholangiocarcinoma (CHOL) (Fig. 3E). *In silico* analysis also revealed the association of *GPR89A* in biological pathways such as intracellular pH reduction, T‐cell activation and differentiation, lymphocyte differentiation, regulation of IκB kinase/NF‐κB signalling and positive regulation of intracellular signal transduction.

### Cisplatin sensitivity increases in *GPR89A* knockout CR‐Calu1 cells

**Fig. 4:**
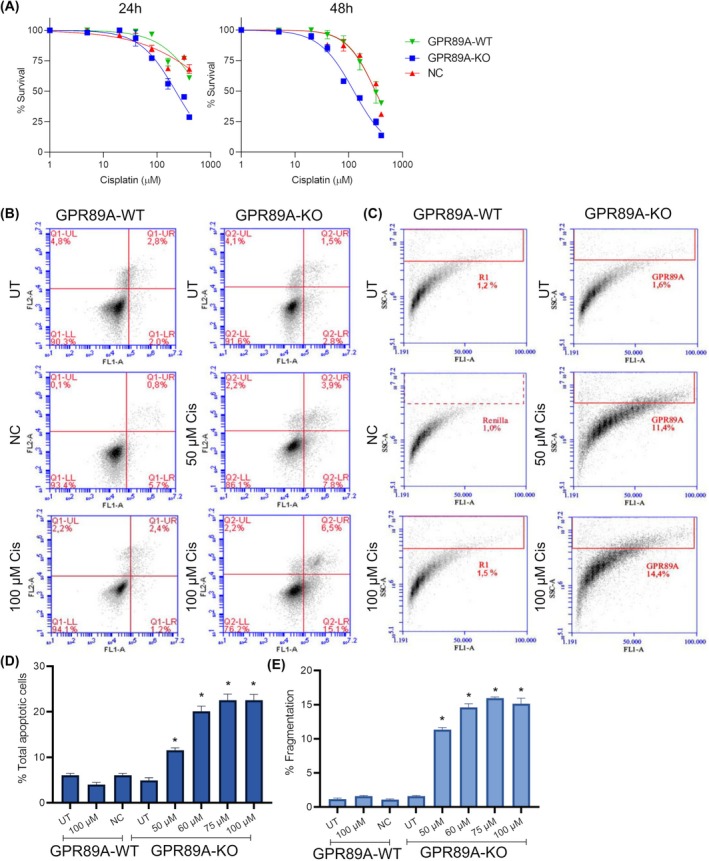
Cytotoxic and apoptotic profiles of *GPR89A* knockout in CR‐Calu1 cells. (A) Cell viability graph of wild‐type CR‐Calu1 (Cisplatin‐Resistant Calu1) (GPR89A‐WT), *GPR89A* knockout CR‐Calu1 (GPR89A‐KO) and *RLuc* gRNA transduced CR‐Calu1 (negative control, NC) cells after 24‐ and 48‐h cisplatin treatment. The IC_50_ doses for cisplatin were determined from the dose–response curves of nonlinear regression analysis using graphpad prism 8.0.2 software. The 24‐ and 48‐h IC_50_ values obtained for the cells were 750.9 and 364.4 μm for WT CR‐Calu1; 235.7 and 123.8 μm for KO CR‐Calu1; and, 743.5 and 352 μm for NC CR‐Calu1 cells, respectively; experiments were performed in triplicates. (B) Flow cytometry images showing the apoptotic effects of cisplatin on GPR89A‐WT, GPR89A‐KO and NC cells according to the Annexin V/PI method; experiments were performed in triplicates (LL, live cell percentage; LR, early apoptotic cell; UL, necrotic cell; UR, late apoptotic cell); (C) flow cytometry images showing the apoptotic effects of cisplatin on GPR89A‐WT, GPR89A‐KO and NC cells according to the TUNEL method; experiments were performed in triplicates; (D) apoptotic fold change graph showing the apoptotic effects of cisplatin on GPR89A‐WT, GPR89A‐KO and NC cells according to the Annexin V method; experiments were performed in triplicates. The statistical analysis was conducted using one‐way ANOVA (*P < 0.0001); (E) DNA fragmentation‐change graph showing the apoptotic effects of cisplatin on GPR89A‐WT, GPR89A‐KO and NC cells according to the TUNEL method; experiments were performed in triplicates. The statistical analysis was conducted using one‐way ANOVA (*P < 0.0001), and results were evaluated with the cflow plus v1.0.264.15 software (error bars indicate standard deviation). NC, CR‐Calu1 cells transduced with the *Renilla reniformis* luciferase (*RLuc*) gRNA carrying lentivirus; UT, untreated cells.

The 24‐ and 48‐h IC_50_ values obtained for cells, treated with cisplatin concentrations ranging from 5 to 400 μm were 750.9 and 364.4 μm for *GPR89A* wild‐type CR‐Calu1 (GPR89A‐WT); 235.7 and 123.8 μm for *GPR89A* knockout CR‐Calu1 (GPR89A‐KO); and 743.5 and 352 μm for 72 *RLuc* gRNA transduced CR‐Calu1 (negative control, NC) cells, respectively (Fig. 4A). Thus, GPR89A‐KO cells were found threefold more sensitive to cisplatin compared to GPR89A‐WT cells, whereas lentivirus transduction with *Rluc* gRNA had no effect on cells.

### Cisplatin induces apoptosis in *GPR89A* knockout CR‐Calu1 cells

Annexin V‐FITC analyses were performed on CR‐Calu1 cells treated with low cisplatin doses ranging from 50 to 100 μm to determine apoptotic cell death levels (Fig. 4B). The apoptotic rate revealed for untreated GPR89A‐WT cells was 4.8%. Accordingly, WT CR‐Calu1 cells were resistant to 100 μm cisplatin concentration (*P* > 0.05). However, the apoptotic rates of GPR89A‐KO cells treated with 50 and 100 μm cisplatin were 11.7% and 21.6%, respectively. Consequently, increased apoptotic rates between these two cisplatin concentrations were significant when compared to untreated GPR89A‐KO cells (*P* < 0.0001). Lentivirus transduction with *Rluc* gRNA (NC) had no effects on the apoptotic rates (Fig. 4B,D).

TUNEL analyses were also performed on CR‐Calu1 cells treated with low cisplatin doses to determine DNA fragmentation, which occurs in late‐stage apoptosis. DNA fragmentation percentages were 1.6% in KO CR‐Calu1, 1.2% in WT CR‐Calu1 and 1% in *RLuc* CR‐Calu1 cells (*P* > 0.05); *GPR89A* KO and lentivirus transduction had no effects on the apoptotic rates obtained. WT CR‐Calu1 cells were cisplatin resistant, since the DNA fragmentation percentage in cells treated with 100 μm cisplatin was 1.5%. However, DNA fragmentation rates increased by 11.4‐fold for 50 μm, 14.8‐fold for 60 μm, 16.1‐fold for 75 μm and 14.4‐fold for 100 μm cisplatin in KO CR‐Calu1 cells when compared to untreated controls (*P* < 0.0001) (Fig. 4C,E). In our previous studies, we reported 7% apoptotic activity in Calu1 cells treated with 13.68 μm cisplatin [17]. Decreased apoptotic activity is one of the mechanisms by which Calu1 cells gain cisplatin resistance [17, 18]. Comparison of Calu1 and *GPR89A* knockout CR‐Calu1 cells shows that the apoptotic activity in knockout cells is restored at the level of parental cells.

### Cisplatin causes cell cycle arrest in *GPR89A* knockout CR‐Calu1 cells

**Fig. 5:**
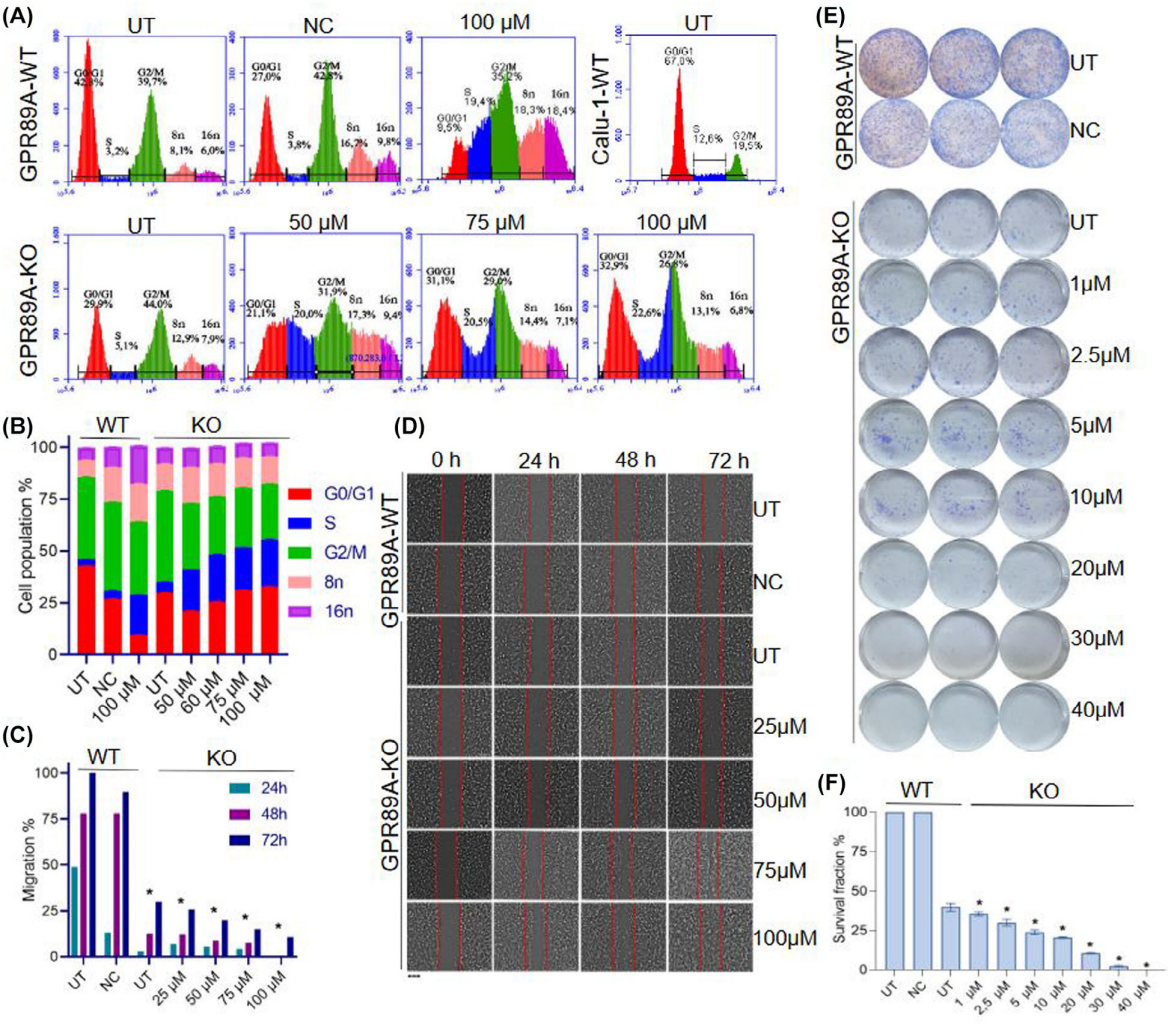
Effects of *GPR89A* knockout in CR‐Calu1 cells on cell cycle, colony formation and cell migration. (A) Flow cytometry images showing the effects of cisplatin on cell cycle in wild‐type CR‐Calu1 (cisplatin‐resistant Calu1) (GPR89A‐WT), GPR89A knockout CR‐Calu1 (GPR89A‐KO) and *RLuc* gRNA transduced CR‐Calu1 (NC) and wild‐type Calu1 (Calu1‐WT) cells; experiments were performed in triplicates; (B) percentages of cell cycle stages affected by cisplatin in GPR89A‐WT, GPR89A‐KO and negative control cells; experiments were performed in triplicates; (C) cell migration rates. The statistical analysis was conducted using one‐way ANOVA (*P < 0.0001); (D) light microscopy migration images in GPR89A‐WT, GPR89A‐KO and negative control cells; experiments were performed in triplicates. Cell migration was measured using the tscratch v.2 software (Scale bar: 500 μm); (E) colony formation assay; experiments were performed in triplicates; (F) survival fraction graph after cisplatin exposure to GPR89A‐WT, GPR89A‐KO and negative control cells. The statistical analysis was conducted using one‐way ANOVA; experiments were performed in triplicates (*P < 0.0001) (error bars indicate standard deviation). NC, CR‐Calu1 cells transduced with the *Renilla reniformis* luciferase ( *RLuc*) gRNA carrying lentivirus; UT, untreated cells.

Cell cycle studies were performed on CR‐Calu1 cells treated with low cisplatin concentrations ranging from 50 to 100 μm. According to the results obtained, the G2/M phase increased from 19.5% in Calu1 to 39% in CR‐Calu1 cells. Interestingly, polyploidy was detected in all cell groups, which led to the conclusion that polyploidy is another mechanism by which Calu1 cells acquire cisplatin resistance. When mitotic phase change rates of UT, WT and *GPR89A* KO CR‐Calu1 cells were analysed, no significant difference was found between their proliferation rates indicating *GPR89A* as not essential for cell proliferation. Treatment of *GPR89A* KO cells with 50–100 μm cisplatin increased the proportion of S‐stage cells from 5% to 20%, causing mitotic catastrophe (Fig. 5A,B).

### Cisplatin inhibits cell migration in *GPR89A* knockout CR‐Calu1 cells

Wound healing analysis was performed on CR‐Calu1 cells treated with low cisplatin concentrations ranging from 25 to 100 μm to determine cell migration rates. After 72 h, the intercellular wounds were fully closed in WT CR‐Calu1 cells, whereas the migration rate of *RLuc* gRNA transduced CR‐Calu1 cells (NC) was 90% (*P* > 0.05); thus, lentivirus transduction itself had no effects on cell migration. In contrast, the 72 h migration rate was 30% in untreated KO CR‐Calu1 cells, and 25% in 25 μm, 20% in 50 μm, 15% in 75 μm and 10% in 100 μm cisplatin‐treated KO CR‐Calu1 cells. Therefore, *GPR89A* KO had an inhibitory effect on cell migration in CR‐Calu1 cells and showed a synergistic effect with cisplatin treatment (Fig. 5C,D).

### Cisplatin inhibits colony formation in *GPR89A* knockout CR‐Calu1 cells

Clonogenic assay was performed on CR‐Calu1 cells treated with low cisplatin concentrations ranging from 1 to 50 μm to determine colony formation. *GPR89A* KO inhibited colony formation, since the survival fraction (SF) of KO CR‐Calu1 cells significantly reduced to 37% when compared to WT CR‐Calu1 and *RLuc* gRNA transduced CR‐Calu1 cells (*P* < 0.0001). In summary, SF for KO CR‐Calu1 cells treated for 48 h was 34.5% for 1 μm, 27.8% for 2.5 μm, 24.3% for 5 μm, 20.6% for 10 μm, 10.4% for 20 μm, 1.9% for 30 μm and 0% for 40–50 μm cisplatin concentrations (*P* < 0.0001). Thus, cisplatin was effective even at low doses and further inhibited colony formation in KO CR‐Calu1 cells (Fig. 5E,F). Comparison of Calu1 and GPR89A‐KO CR‐Calu1 cells showed that colony formation in Calu1 cells decreased significantly at 50 μm cisplatin, and colonies did not form at subsequent increasing doses (Fig. 1B). Survival fractions of Calu1 and *GPR89A*‐KO CR‐Calu1 cells were 16% and 75%, respectively.

## Discussion

Lung cancer is the second most common cancer type in humans and the leading cause of cancer‐related deaths, mainly due to its rapid progression and high metastatic potential [19]. The estimated number of newly diagnosed lung cancer cases and cancer‐related deaths for the United States in the year 2023 are 238.340 and 127 070, respectively. Its 5‐year survival rate after diagnosis, on the other hand, is 15.6% and lower than for breast, prostate and colon cancer cases [20]. Among all cancer types affecting the pulmonary system, NSCLC leads with ~ 85% and standard therapy of NSCLC cases includes chemotherapy with the platinum‐based chemotherapeutic agent cisplatin [21]. Although NSCLC cases respond well to cisplatin at the beginning of their treatment, ~ 60% of them develop chemotherapy resistance during this process [22]. Drug resistance can either arise from intrinsic or acquired resistance and cancer cells do not only become resistant to the medications employed in their treatment but also develop cross‐resistance to other medications with distinct modes of action [23, 24, 25]. Despite which way cancer cells gain drug resistance, they all lead to reduced treatment efficacy and have a negative impact on the therapeutic process [26]. Thus, identifying targetable genetic components responsible for cisplatin resistance will help to improve existing cancer therapies and overcome such throwbacks.

Lately, CRISPR‐Cas9‐based genetic screenings have been employed to identify genes that cancer cells are depending on, or are addicted to, in association with cisplatin resistance [27]. In a kinome, CRISPR‐Cas9 knockout screen in which kinases in malignant pleural mesothelioma (MPM) cancer cells were targeted *WEE1*, *AURKA*, *MPP3*, *MAP3K12*, *DGKD* and *SPEG* could be identified as candidate genes. Further validation studies for the *WEE1* gene, that encodes a G_2_‐M checkpoint kinase, revealed that loss of its function sensitises MPM cells to the standard pemetrexed (MTA)/cisplatin chemotherapy [28]. In a genome‐wide CRISPR‐Cas9‐based genetic screening study carried out with the GeCKO library in two bladder cancer cell lines, *MSH2*, *MLH1*, *FAM89B*, *XPC* and *PMS2* were identified as the top five candidate genes. Further studies for *MSH2*, a mismatch repair protein‐encoding gene, showed that its low protein expression level may contribute to cisplatin resistance observed in muscle‐invasive bladder cancer [29]. In a similar study with the GeCKO library in the A2780 and SKOV3 ovarian cancer cell lines, *ZNF587B*, *TADA1*, *SEMA4G*, *POTEC* and *USP17L20* were identified as candidate genes; among these, loss of *ZNF587B* and *SULF1* gene expressions were associated with cisplatin resistance [30]. However, in another CRISPR‐Cas9‐based genetic screening study in the Kuramochi and OVSAHO ovarian cancer cell lines the anti‐apoptotic genes *BCL2L1* (BCL‐XL) and *BCL2L2* (BCL‐W) were found to be associated with chemotherapy resistance [31]. Small hairpin RNA (shRNA) and genome‐wide CRISPR‐Cas9‐based genetic screenings in melanoma cells, on the other hand, revealed *ZNRF3*, *NF2* and *ARIH1* as genes associated with cisplatin resistance [32]. Screening of the HAP1 haploid human cell line with a CRISPR‐Cas9‐library targeting 388 solute carrier (SLC) transmembrane transporter genes revealed that the SLC35A2/SLC38A5 transmembrane transporters play an important role in cisplatin resistance [33]. Thus, CRISPR‐Cas9‐based genetic screening studies carried out with different libraries, cancer types and cell lines identified different candidate genes involved in cisplatin resistance. In this current study, a genome‐wide CRISPR‐Cas9‐based genetic screening with the Brunello CRISPR knockout library was carried out for the first time in cisplatin‐resistant NSCLC Calu1 cells that identified *GPR89A*, *PUF60*, *NBAS* and *GRPEL1* as novel candidate genes. Although recently a genome‐wide CRISPR‐Cas9‐based genetic screening study was carried out with the GeCKO library in the NCI‐H460, A549 and NCI‐H1299 human NSCLC cell lines that identified *MDM2*, a nuclear‐localised E3 ubiquitin ligase encoding gene, as a potential therapeutic target, these cells were not cisplatin resistant [34].

*GPR89A* has been shown in association with cisplatin resistance in Calu1 cells for the first time in this study. It localises on chromosome 1q21.1 and encodes for the G protein‐coupled receptor 89A located in the Golgi cisterna and Golgi‐associated vesicle membrane. *GPR89A* forms a voltage‐gated chloride (Cl^−^) channel that regulates pH of the Golgi through ion conductance and also controls post‐translational modifications of the plasma membrane and secretory proteins [35]. Its inactivation or decreased expression results in structural disarray of the Golgi, impaired glycosylation of proteins and a delayed transfer of freshly synthesised proteins from the Golgi to the plasma membrane [36]. In recent years, the importance of pH homeostasis in cancer progression has gained attention. Cancer cells usually demand higher levels of energy than normal cells for growing and surviving, which they obtain through increased glucose uptake. The energy gained through glycolysis leads to the formation of an alkaline pH and inhere Golgi functions as a proton pool, contributing to the maintenance of this alkaline cytosolic pH [36, 37]. The involvement of *GPR89A* in different cancer types has also been reported lately, for example repression by siRNA application resulted in increased apoptotic death rates of PC3 and DU145 prostate cancer cells. When *GPR89A* siRNAs were applied in combination with the chemotherapeutic agent docetaxel, they even caused a synergistic effect. Since *GPR89A* was not mutated *per se* in these prostate cancer cells, it was probably a synthetic lethal gene partner of a specific oncogene [38]. In this current study, *GPR89A* gene knockout by CRISPR‐Cas9 in combination with cisplatin exposure in CR‐Calu1 cells also caused a synergistic effect by increasing apoptotic cell death rates of NSCLC cells. High *GPR89A* gene expression levels in aggressive papillary adenocarcinoma and amplification of the chromosomal 1q21‐43 region, including the PI4KIIIβ and *GPR89A* genes, in lung cancer cells have been shown to be associated with tumour progression and metastasis [39]. In breast cancer cells, *GPR89A* has been identified as a novel oncogene cooperating with the *MYC* oncogene to promote tumour formation. When its increased expression was inhibited, it impaired cell proliferation and colony‐forming abilities of breast cancer cells [40]. Interestingly, *GPR89A* was also found amplified and highly expressed across breast cancer subtypes in a cohort of another study. Functional analyses revealed that its overexpression is important in breast cancer cell proliferation, and besides the Golgi, GPR89A also localises in the endoplasmic reticulum (ER) [41]. In the ER, GPR89A has been shown to negatively regulate the ATF4/CHOP pathway and positively regulate the IRE1/XBP1 pathway as an unfolded protein response (UPR) to ER stress [41]. Breast cancer cells undergo significant stress due to accelerated metabolism, deficient nutrient provision and oxidative stress in order to sustain their uncontrolled growth. Therefore, *GPR89A* overexpression and UPR signalling are essential mechanisms to protect cancer cells and allow their growth by reducing protein synthesis and favouring chaperonin functions [42]. Since in this current study *GPR89A* gene knockout has also resulted in reduced cell proliferation, migration and colony formation, it might be used as a new therapeutic target in combination with chemotherapy to overcome cisplatin resistance in NSCLC cells. Given the limited number of studies investigating the role of *GPR89A* in cancer, its mechanism of action and relationship with cisplatin resistance remain poorly understood. To address this, further studies employing transcriptome analysis, three‐dimensional cell culture and different model organisms are required.

In conclusion, genome‐wide CRISPR‐Cas9‐based genetic screening identified 63 candidate genes responsible for cisplatin resistance in Calu1 cells. *GPR89A* has been found as one of the candidate genes, and its knockout in CR‐Calu1 cells resulted in increased apoptosis and polyploidy and prevented colony formation and cell migration. Cisplatin treatment of these cells, on the other hand, resulted in increased apoptosis due to the cell cycle arrest in the S phase. In conclusion, this is the first study that identified *GPR89A* as a potential therapeutic target to overcome cisplatin resistance in NSCLC cells.

## Materials and methods

### Cell culture

The cisplatin‐sensitive NSCLC cell line Calu1 (RRID: CVCL_0608) was used to generate cisplatin‐resistant‐Calu1 (CR‐Calu1) cells by the dose escalation method, as previously described [17]. In brief, at the beginning Calu1 cells were exposed to 5 μm cisplatin to establish cisplatin‐resistant sublines. After 24 h, the medium was aspirated and cells were grown in a normal medium for 2 weeks. Resistant cells from each subculture were exposed to 1.5‐fold concentrations of cisplatin at each dose increase for 1 year, until Calu1 cells were treated with a final dose of 250 μm cisplatin. Parental Calu1 cells were also cultured in parallel for the same time period. These cells were then used in genome‐wide CRISPR‐Cas9‐based genetic screening, knockout and functional studies. Meanwhile, the human embryonic kidney cell line 293T cells (HEK293T; RRID: CVCL_0063) were used for lentivirus production and packaging. Calu1 and HEK293T cells were grown in DMEM medium supplemented with 10% FBS, 1% penicillin–streptomycin and 1% l‐glutamine (Biological Industries, Sartorius, Gottingen, Germany) and incubated at 37 °C with 5% CO_2_ and 95% humidity. Ampicillin (A0166; Sigma‐Aldrich, St. Louis, MO, USA ) 100 mg·mL^−1^ stock concentrations were prepared and stored at −20 °C together with cisplatin (13119; Cayman Chemical, Ann Arbor, MI, USA), blasticidin (ant‐bl‐05; InvivoGen, San Diego, CA, USA) and puromycin (ant‐pr‐1; InvivoGen, San Diego, CA, USA). All cell lines have been authenticated in the past 3 years by morphological validation and STR profiling. Cells were regularly tested for mycoplasma contamination by Hoechst staining.

### Cytotoxicity assays

Cytotoxicity assays were performed to determine cytotoxic effects of cisplatin on Calu1, wild‐type (WT) and *GPR89A* knockout (KO) CR‐Calu1 cells, and for the evaluation of cytotoxic inhibitory concentrations that kill 50% of cells (IC_50_). In brief, Calu1 (5 × 10^3^ cells/well), WT and KO CR‐Calu1 (6 × 10^4^ cells/well) cells were seeded in 96‐well plates and incubated for 24 h. After incubation, Calu1, WT and KO CR‐Calu1 cells were treated with different cisplatin concentrations in triplicates. Cisplatin‐untreated cells and cells transduced with the *Renilla reniformis* luciferase (*RLuc*) gRNA, with no target gene in the human genome, were also used and considered as control and negative control groups, respectively. After incubation for 24, 48 and 72 h, XTT solution was added into each well. The qualitative value of formazan dye formation was measured photometrically at 450 and 620 nm by using the Multiskan FC ( Thermo Fisher Scientific, Waltham, MA, USA) microplate reader. The graphpad prism 8.0.2 (GraphPad Software, Inc. Boston, MA, USA) analysis programme was used to calculate IC_50_ cytotoxic doses for cisplatin.

### Lentiviral vectors and library

In this study, the LentiCas9‐Blast (52962; Addgene, Watertown, MA, USA) vector for stable Cas9 expression in Calu1 and CR‐Calu1 cells; lentiGuide‐Puro (52963; Addgene, Watertown, MA, USA) vector for cloning target specific gRNAs; and LentiCRISPR V2 (52961; Addgene, Watertown, MA, USA) vector for expressing both Cas9 and gRNA were used. Cells were selected with blasticidin when transduced with the LentiCas9‐Blast vector and with puromycin when transduced with either the LentiGuide‐Puro or LentiCRISPR V2 vectors. Also, the lentiviral ‘Human CRISPR Brunello pooled library (73178; Addgene, Watertown, MA, USA)’ was used in this study consisting of a total of 76 441 guide RNAs (gRNAs) targeting 19 114 protein‐coding genes (4× gRNAs/gene) and 1000 control gRNAs with no target gene in the human genome (73178; Addgene, Watertown, MA, USA).

### Lentivirus production and titration

Lentivirus production was accomplished in HEK293T cells transduced with the psPAX2 (12260; Addgene, Watertown, MA, USA) vector expressing the *gag* and *pol* genes, and the pMD2.G (12259; Addgene, Watertown, MA, USA) vector expressing the *VSV‐G* envelope protein gene. Optimal virus titrations were identified by seeding HEK293T cells in 6‐well plates (7 × 10^5^ cells/well) and incubating them at 37 °C for 24 h. Cells were then transfected with the LentiCas9 Blast, LentiGuide‐Puro or LentiCRISPR V2 : psPAX2 : pMD2.G (4 : 2 : 1 mix ratio) vectors in 100 μL PBS. Meanwhile, the DNA mixture was added into 12.5 μL polyethylenimine (23966; Polysciences Inc., Warrington, PA, USA) containing 100 μL PBS and added to HEK293T cells in the presence of fresh medium when reaching 80% cell density. After 48 h of transfection, the lentivirus‐containing media was collected from the culture dishes, purified by a 0.45 μm SFCA filter, aliquoted and stored at −86 °C until use. For the determination of multiplicity of virus infection (MOI) rates, 3 × 10^5^ cells/well Calu1 or 4 × 10^5^ cells/well CR‐Calu1 cells were seeded into 6‐well plates and incubated at 37 °C for 24 h. Lentivirus dilutions of 1/16, 1/32, 1/64 and 1/128 were added onto the cells along with polybrene (8 mg/mL) (9268; Sigma‐Aldrich, St. Louis, MO, USA) to increase transduction efficiency. After 48 h of transduction, the cells were washed with PBS and then selected with the appropriate doses and durations of antibiotics. At the end, the number of remaining cells were compared with untransduced and unselected control cells. For genome‐wide CRISPR‐Cas9‐based genetic screening experiments and validation studies, lentivirus concentrations were used with 30% (MOI = 0.3) and 50% (MOI = 0.5) transduction efficiencies, respectively.

### Cas9 expressing Calu1 and CR‐Calu1 cells

2 × 10^6^ cells of Calu1 and CR‐Calu1 were seeded in T‐150 flasks and incubated at 37 °C for 24 h. After incubation, Calu1 and CR‐Calu1 cells were transduced with the appropriate lentivirus titration (1/20 and 1/100, respectively) with polybrene and fresh medium (Fig. S1A). After 48 h, cells expressing the Cas9 enzyme were selected with blasticidin for 9 days and propagated further for another 14 days with normal medium (Fig. S1B). Proteins were purified with the cOmplete™ Lysis‐M reagent (4719956001; Roche, Basel, Switzerland) and quantified with the Bradford method before western blot analyses were performed. Anti‐FLAG (1 : 1000; F1804; Sigma‐Aldrich, St. Louis, MO, USA ) antibody was used to show Cas9 expression and anti‐β‐actin (1 : 1000; 4970; Cell Signaling Technology, Danvers, MA, USA) antibody was used as a loading control. Cas9 functionality was also proofed by western blot analyses in cells transduced with either the epidermal growth factor receptor (EGFR) (1 : 1000; anti‐EGFR; 2232S; Cell Signaling Technology, Danvers, MA, USA) or *RLuc* gRNA containing lentiGuide‐Puro vectors.

### Genome‐wide CRISPR‐Cas9‐based genetic screening

Genome‐wide CRISPR‐Cas9‐based genetic screening studies were initiated with 255 × 10^6^ cells for 1000× representation of each gRNA in the lentiviral Brunello CRISPR knockout library. In brief, *Cas9*‐expressing Calu1 and CR‐Calu1 cells were seeded into 85 × 150 mm plates (3 × 10^6^ cells/plate) and incubated at 37 °C for 24 h. After incubation, cells were transduced with the appropriate lentivirus titration (MOI = 0.3, 1/150) and incubated for another 48 h (Fig. S1C). Afterwards, gRNA‐expressing cells were selected for 3 days with puromycin (1 μg/mL) (Fig. S1D). Following selection, total cell numbers were evaluated and 8 × 10^7^ cells were stored at −86 °C for subsequent genomic DNA isolation, designated as Calu1 T0 and CR‐Calu1 T0, and later used for the determination of reference gRNA distribution. Genetic screening was performed after Calu1 and CR‐Calu1 cells were divided into cisplatin‐treated and cisplatin‐untreated groups, each with 8 × 10^7^ cells/group and studied in triplicates. Whereas cisplatin‐untreated cell groups were grown in normal media, cisplatin‐treated cell groups were given the cisplatin dose killing 20% of cells after 7 days of transduction and incubated for another 48 h. After incubation, genomic DNA was isolated from all cell groups by using the NucleoSpin Blood XL Kit (Macherey‐Nagel, Duren, Germany).

### Library preparation and sequencing

All isolated genomic DNAs were labelled with index primers before libraries were created for each of the 14 cell groups. In brief, forward and reverse primers (P5 and P7) were used to amplify gRNAs (Tables S1 and S2). Eight different P5 primers were pooled and used to minimise reading errors during sequencing. For library preparation, genomic DNA was amplified with the Q5^®^ High‐Fidelity DNA Polymerase (M0491S; NEB, Ipswich, MA, USA). The reaction mixture for PCR was prepared using 50 μg of genomic DNA with 200 μL of 5× Q5 Reaction Buffer, 20 μL of dNTP (10 mm), 50 μL of Forward Primer (10 μm), 50 μL of Reverse Primer (10 μm), 10 μL of Q5 High‐Fidelity DNA Polymerase, 200 μL of 5× Q5 High GC Enhancer and up to 1000 μL nuclease‐free water. The applied thermal cycling protocol was initial denaturation at 98 °C for 30 s; 28 cycles of amplification (10 s at 98 °C, 30 s at 53 °C and 30 s at 72 °C); and a final extension step at 72 °C for 2 min. After loading and running the PCR products on a 2% agarose gel, the index‐labelled 317 bp DNA band was cut out and purified with the NucleoSpin Gel and PCR Clean‐up Kit (Macherey‐Nagel, Duren, Germany) (Fig. S1E). Each sample was subjected to quality control (QC) analysis, before being pooled and next‐generation sequencing (NGS) analysis performed on the Illumina HiSeq X Instrument (Macrogen, Inc., Seoul, South Korea). Sequencing was performed by reading 150 bp covering the gRNA region and 8 bp corresponding to the index. 30% PhiX control DNA was utilised in NGS for lowering reading errors.

### Screening data analyses

fastq files were analysed with the MAGeCK algorithm to determine the number of gRNA reads/group and normalised to the initial T0 groups [43]. After normalisation, depleted and enriched gene scores were calculated for each group. Candidate genes responsible for cisplatin resistance were selected according to neglfc < −1, good gRNA number ≥ 3 and *P* < 0.05‐fold change ratios. Among these negatively selected genes, the candidate *GPR89A* gene was further evaluated by functional studies.

### *GPR89A* knockout CR‐Calu1 cells

The gRNA sequence (i.e., CGGAGAACAATGTTCCAGAA) with the highest efficiency from the NGS data was selected and cloned into the LentiCRISPR V2 vector to generate *GPR89A* knockout CR‐Calu1 cells. After confirmation by Colony PCR and Sanger sequencing, the vector was packaged into lentiviruses before transducing CR‐Calu1 cells (MOI = 0.5) (Fig. S1F,G). After transduction, cells were selected with puromycin for 3 days before growing in normal medium. *GPR89A* knockout was validated by the Sanger sequencing method. In brief, specific primers up‐ and downstream the gRNA region were used to detect indel mutations introduced by the nonhomologous end joining (NHEJ) DNA repair mechanism (forward: GAGGCAGTTGTGAGACTGGA, reverse: TCAAGCAGAGGGACAAAGGAT; gRNA: CGGAGAACAATGTTCCAGAA). DNA was isolated from CR‐Calu1 and *GPR89A* knockout CR‐Calu1 cells and Sanger sequencing was performed with 5′ → 3′ and 3′ → 5′ reads. As a result, indel mutations were shown in *GPR89A* knockout CR‐Calu1 cells (Fig. S2A,B).

### Apoptosis and cell cycle analyses

Apoptosis analyses were carried out by using the BD Pharmingen™ FITC Annexin V Apoptosis Detection Kit II and ApoDIRECT *In Situ* DNA Fragmentation Assay Kit (BioVision, Milpitas, CA, USA). Cell cycle analyses were carried out by using the BD Cycletest™ Plus DNA Reagent Kit. In brief, 1.8 × 10^5^ cells/well were seeded in 6‐well plates and incubated at 37 °C for 24 h. After incubation, cells were treated with different cisplatin concentrations for 48 h. Untreated CR‐Calu1 and *RLuc *gRNA transduced CR‐Calu1 cells were used as control groups. After flow cytometric analyses on the BD Accuri‐C6 instrument (BD Biosciences, San Jose, CA, USA), results were evaluated with the cflow plus v1.0.264.15 software (Accuri Cytometers Inc., Ann Arbor, MI, USA).

### Colony formation

Clonogenic assays were performed to determine the long‐term effects of cisplatin on resistant cells. For this purpose, 1 × 10^3^ cells/well were seeded in 6‐well plates and incubated at 37 °C for 24 h. After incubation, cells were treated with different cisplatin concentrations for 48 h. Untreated CR‐Calu1 and *RLuc* gRNA transduced CR‐Calu1 cells were used as control groups. After incubation, cells were washed with PBS and cultured for another 14 days in normal medium, fixed with cold MeOH, stained with toluidine blue and counted under a stereomicroscope. Finally, coating efficiency and survival fraction were calculated for each cisplatin dose.

### Cell migration

Wound healing analyses were performed to determine the effects of cisplatin on cell migration. For this purpose, 2 × 10^5^ cells/well were seeded in 6‐well plates and incubated at 37 °C for 24 h. After incubation, the media was removed and an intercellular wound in each well was created by means of a pipette tip. Finally, cells were treated with different cisplatin concentrations. Untreated CR‐Calu1 and *RLuc* gRNA transduced CR‐Calu1 cells were used as control groups. Images were taken after 0, 24, 48 and 72 h, and migration abilities of cells were measured by the relative open area using the tscratch v.2 software (CSE Lab, Zurich, Switzerland) and normalised to the initial time.

### Bioinformatics and statistical analyses

The Metascape database is an important web resource for high‐throughput gene function analyses, that is used to obtain functional and pathway enrichment annotations of candidate genes [44]. Top 20 Kyoto Encyclopedia of Genes and Genomes (KEGG) and Gene Ontology (GO) terms were selected to be represented in the graphs. UALCAN, on the other side, is a comprehensive web resource and provides analyses of candidate genes based on The Cancer Genome Atlas (TCGA) [45]. Expression data for *GPR89A* were obtained using the Expression Analysis module of UALCAN and the LUSC dataset. *GPR89A* expression levels were further analysed by using the UALCAN database, as well as boxplots, stage plots and survival analyses. Student's *t*‐test and ANOVA tests were used in graphpad prism 8.0.2 (GraphPad Software, Inc. Boston, MA, USA) analysis programme for comparative statistical analysis (mean ± SD) of the cell groups. The *P*‐value cut‐off was set as 0.05.

## Conflict of interest

The authors declare no conflict of interest.

## Author contributions

HGK, BK and VB designed the experiments; HGK, ED, DAJ and EC performed the experiments; HGK, BK, VB, CA, KSK and SS analysed the data; HGK, BK and VB wrote the manuscript; ZE, KSK, SS, BK and VB revised the manuscript; all authors read and approved the final version of the manuscript.

## Peer review

The peer review history for this article is available at https://www.webofscience.com/api/gateway/wos/peer‐review/10.1111/febs.70099.

## Supporting information


**Fig. S1.** CRISPR‐Cas9‐based genetic screening and cloning studies.
**Fig. S2.** Indel mutation screening for knockout validation of the GPR89A gene in CR‐Calu1 cells.
**Table S1.** P5 primers.
**Table S2.** P7 barcode (index) sequences.

## Data Availability

The data related to the study are included in the figure, text and Supporting Information of the article. Further data are available on request from the corresponding author.
