# Supplementary material for: The G protein‐coupled receptor GPR89A is a novel potential therapeutic target to overcome cisplatin resistance in NSCLC Calu1 cells
Source: FEBS J. 2025 Apr 17;292(14):3755–70. doi: 10.1111/febs.70099 (PMC12265858; doi:10.1111/febs.70099)
Supplement: Supplementary file 1 — Fig. S1. CRISPR‐Cas9‐based genetic screening and cloning studies. Fig. S2. Indel mutation screening for knockout validation of the GPR89A gene in CR‐Calu1 cells. Table S1. P5 primers. Table S2. P7 barcode (index) sequences. [file FEBS-292-3755-s001.pdf]

The G protein-coupled receptor GPR89A is a novel potential therapeutic target to overcome cisplatin resistance in NSCLC Calu1 cells

Supplemantary Figures and Tables

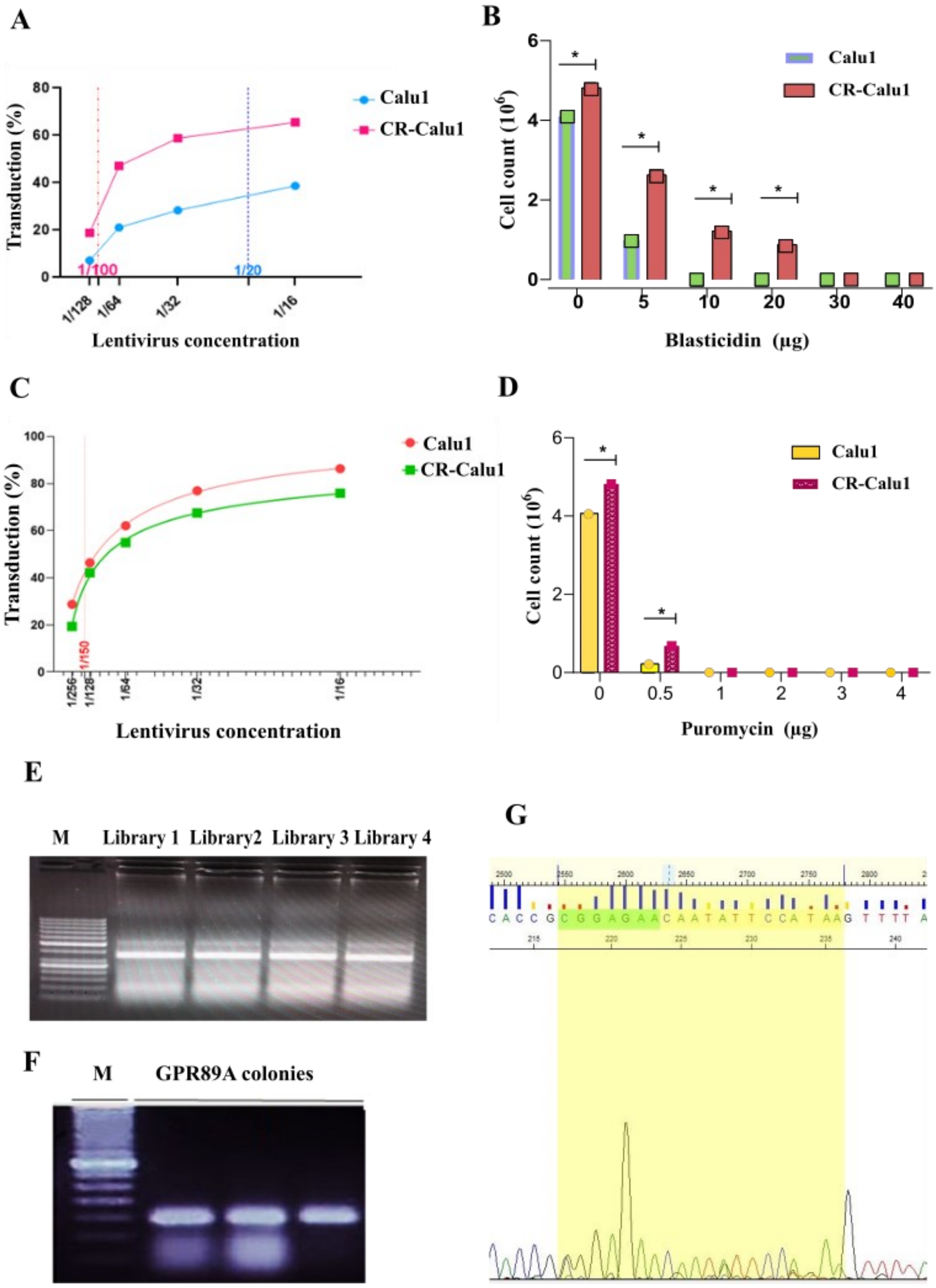

Fıgure 1: CRISPR-Cas9-based genetic screening and cloning studies. A) Transduction percentages according to the concentrations of LentiCas9 Blast lentivirus to be used to infect Calu1 and CR-Calu1 cells (MOI=0.3); B) Blasticidin concentrations required for the selection of Cas9-expressing Calu1 and CR-Calu1 cells ($p<0,0001$); C) Transduction percentages according to the concentrations of LentiGuide Puro lentivirus to be used to infect Cas9 expressing-Calu1 and CR-Calu1 cells (MOI=0.3); D) Puromycin concentrations required for the selection of *gRNA*-expressing Calu1 and CR-Calu1 cells ($p<0,0001$); E) Amplification and indexing of libraries generated after CRISPR-Cas9 genetic screening with primers P5 and P7 in Calu1 and CR-Calu1 cells [M: Marker (100bp) Library 1: CR-Calu1-T0, Library 2: CR-Calu1-UT1, Library 3: CR-Calu1-UT2, Library 4: CR-Calu1-UT3]; F) Colony PCR and G) Sanger sequencing following cloning of the GPR89A gene *gRNA* into the LentiCRISPR V2 plasmid for validation of cloning.

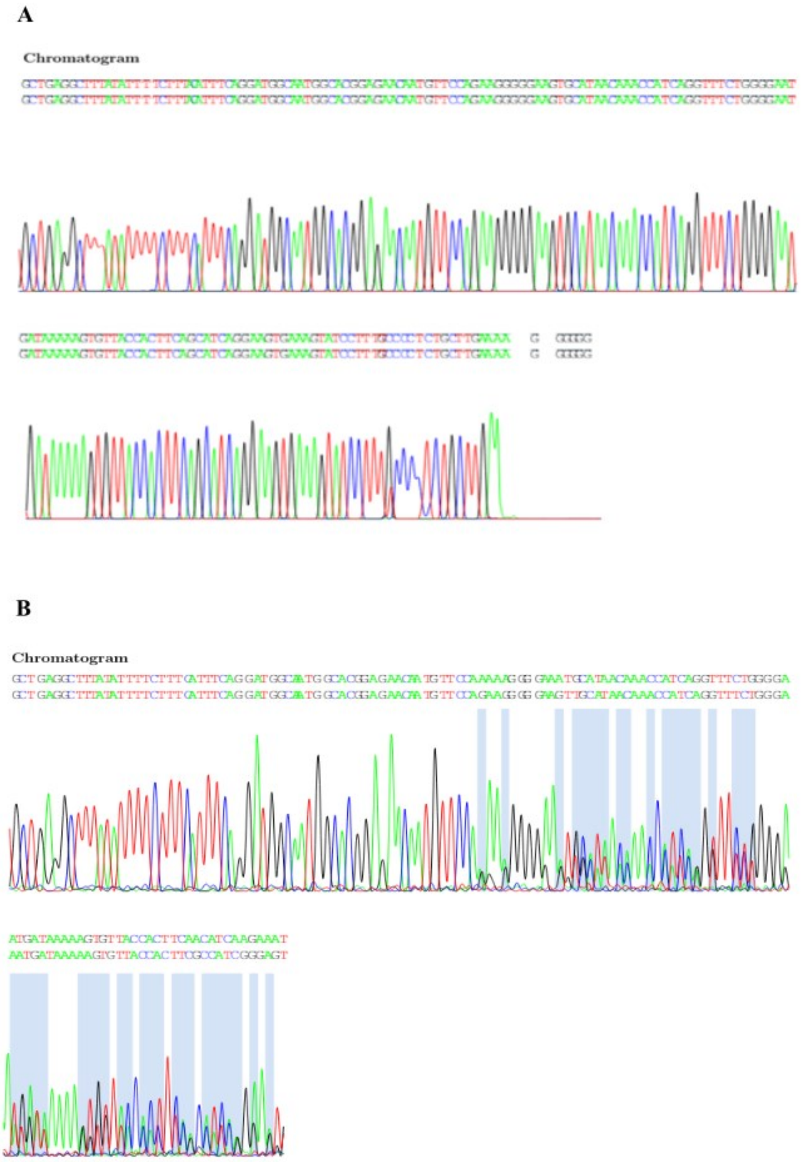

Figure 2: Indel mutation screening for knockout validation of the *GPR89A* gene in CR-Calu1 cells.

Sanger sequencing of parental (A) and GPR89A knockout CR-Calu1 cells (B) for knockout validation

Table 1: P5 Primers

P5 primers	
**Name**	**Sequence**
P5 0 nt stagger	AATGATACGGCGACCACCGAGATCTACACTCTTTCCCTACACGACGCTCTTCCGATCTTTGTGGAAAGGACGAAACACCG
P5 1 nt stagger	AATGATACGGCGACCACCGAGATCTACACTCTTTCCCTACACGACGCTCTTCCGATCTCTTGTGGAAAGGACGAAACACCG
P5 2 nt stagger	AATGATACGGCGACCACCGAGATCTACACTCTTTCCCTACACGACGCTCTTCCGATCTGCTTGTGGAAAGGACGAAACACCG
P5 3 nt stagger	AATGATACGGCGACCACCGAGATCTACACTCTTTCCCTACACGACGCTCTTCCGATCTAGCTTGTGGAAAGGACGAAACACCG
P5 4 nt stagger	AATGATACGGCGACCACCGAGATCTACACTCTTTCCCTACACGACGCTCTTCCGATCTCAACTTGTGGAAAGGACGAAACACCG
P5 6 nt stagger	AATGATACGGCGACCACCGAGATCTACACTCTTTCCCTACACGACGCTCTTCCGATCTTGCACCTTGTGGAAAGGACGAAACACCG
P5 7 nt stagger	AATGATACGGCGACCACCGAGATCTACACTCTTTCCCTACACGACGCTCTTCCGATCTACGCAACTTGTGGAAAGGACGAAACACCG
P5 8 nt stagger	AATGATACGGCGACCACCGAGATCTACACTCTTTCCCTACACGACGCTCTTCCGATCTGAAGACCCTTGTGGAAAGGACGAAACACCG

(green: P5/P7 flowcell attachment sequence, blue: Illumina sequencing primer, purple: Stagger region / Barcode region, red: Vector primer binding sequence)

Table 2: P7 barcode (index) sequences

Name	Sequence to include in P7 primer, 5'-3'	Index read (reverse Complement of sequence)
A01	CGGTTCAA	TTGAACCG
A02	GCTGGATT	AATCCAGC
A03	TAACTCGG	CCGAGTTA
A04	TAACAGTT	AACTGTTA
A05	ATACTCAA	TTGAGTAT
A06	GCTGAGAA	TTCTCAGC
A07	ATTGGAGG	CCTCCAAT
A08	TAGTCTAA	TTAGACTA
A09	CGGTGACC	GGTCACCG
A10	TACAGAGG	CCTCTGTA
A11	ATTGTCAA	TTGACAAT
A12	TATGTCTT	AAGACATA
B01	ATTGGATT	AATCCAAT
B02	ATACTCGG	CCGAGTAT
